# From Doxastic to Epistemic: A Typology and Critique of Qualitative Interview Styles

**DOI:** 10.1177/1077800418810724

**Published:** 2018-11-28

**Authors:** Astrid Berner-Rodoreda, Till Bärnighausen, Caitlin Kennedy, Svend Brinkmann, Malabika Sarker, Daniel Wikler, Nir Eyal, Shannon A. McMahon

**Affiliations:** 1 Heidelberg University, Baden-Württemberg, Germany; 2 Johns Hopkins University, Baltimore, MD, USA; 3 Aalborg University, Denmark; 4 BRAC University, Dhaka, Bangladesh; 5 Harvard T.H. Chan School of Public Health, Boston, MA, USA

**Keywords:** qualitative interviews, interview styles, doxastic, epistemic, deliberation

## Abstract

Qualitative interview styles have been guided by precedent within academic disciplines. The nature of information sought, and the role of interviewer and interviewee are key determinants across styles, which range from doxastic (focused on understanding interviewees’ experiences or behaviors) to epistemic (focused on co-constructing knowledge). In this article, we position common interview styles along a doxastic–epistemic continuum, and according to the role of the interviewee (from respondent to equal partner). Through our typology and critique of interview styles, we enhance epistemic interviewing by introducing “deliberative interviews,” which are more debate oriented and closer to equality in the interviewee and interviewer relationship than existing interview styles. Deliberative interviews require a comprehensive, pre-interview briefing on the subject matter followed by interactive deliberation wherein complex issues are debated across viewpoints in an effort to devise solutions. The effectiveness of this interview style in generating new knowledge warrants empirical testing across academic disciplines.

## Introduction

Qualitative interviews are an integral part of many, if not most, qualitative research studies, particularly in the fields of sociology, social anthropology, psychology, education, and public health (Brinkmann, 2007; DiCicco-Bloom & Crabtree, 2006; Kvale, 2007; Qu & Dumay, 2011; Seidman, 1998; Spradley, 1979). While a variety of styles exist across academic disciplines in terms of the nature and content of a qualitative interview (DiCicco-Bloom & Crabtree, 2006, p. 314) and the interaction between interviewee and interviewer^1^ (Cassell, 1980, p. 29; Corbin & Morse, 2003, p. 340), several consistencies exist including the planning, which precedes an interview (Creswell, 2013, pp. 163-166), the time-frame for an interview, and the use of pre-defined and probing questions to uncover or clarify insights. A qualitative interview, like qualitative research generally, is focused on “depth rather than breadth” (Rubin & Rubin, 2011, p. 2), and seeks to capture attitudes and lived experiences of interviewees while unearthing or generating knowledge (Bernard, 2006; Brinkmann, 2007; Seidman, 1998; Ulin, Robinson, Tolley, & McNeil, 2005; Weiss, 1995).

Our analysis takes Brinkmann’s (2007) distinction between “doxastic interviews” (which focus on understanding interviewees’ experiences or behaviors) and “epistemic interviews” (which focus on co-constructing knowledge through the interaction between the interviewee and the interviewer) as the starting point. We then construct a typology of interviews based on the doxastic–epistemic distinction, and a related but separate distinction based on the role of the interviewee. That role can range from a respondent who answers questions posed by the interviewer to an equal partner who queries arguments and challenges the interviewer in a debate about a topic of interest. With increasing focus on epistemic purpose, the relationship between interviewee and interviewer tends to resemble an equal partnership. Yet we also point out that while the correlation of epistemic and equal partnership exists, there are doxastic interviews that show elements of equality in the interview relationship (McNair, Taft, & Hegarty, 2008; Oakley, 1981), and there are epistemic interviews that do not seem to be based on equal relationships between interviewer and interviewee (Bellah, Madsen, Sullivan, Swidler, & Tipton, 2008).

We describe and critique five doxastic and four epistemic interview styles and position them along the two continua of doxastic-to-epistemic purpose and interviewee–interviewer relationship from respondent to equal partner. We then introduce and develop a novel interview style, the deliberative interview, based on a research method need.^2^ Deliberative interviews are both more epistemic in purpose and closer to equality in the relationship between interviewee and interviewer than existing interview styles. This deliberative style, like group deliberation methods, will require a comprehensive pre-interview briefing on the interview subject matter to enable interactive deliberation. Interviewee and interviewer will debate issues from different angles in an effort to devise solutions to complex questions. We regard the deliberative interview style, therefore, of relevance to various academic disciplines.

## Historical Perspective

Qualitative interviews were employed in the early 20th century by social anthropologists (Malinowski, 1922; Mead, 1959) yet became more established as a research method across academic fields including sociology, psychology, and education in the 1970s and 1980s. In many respects, qualitative research was borne out of a recognition that positivist, quantifiable methods were insufficient to understand human behavior and capture social and cultural realities (Kvale, 2006; Seidman, 1998). However, it was the 1980s “wars” (Denzin, 2009, p. 14) between quantitative and qualitative research “paradigms”^3^ that shaped the way qualitative interviews are commonly conducted.

Qualitative interviews are often seen as a product of and counter-movement to experimental studies of subjects, particularly in psychology and in education (Kvale, 2006, p. 481; Seidman, 1998, pp. xviii-xix), where personal encounters and interactions superseded experiments with subjects. “In contrast to such alienated relations of researcher and subjects, dialogue suggested mutuality and egalitarianism; with their gentle, unassuming, nondirective approaches, qualitative interviewers entered into authentic personal relationships with their subjects” (Kvale, 2006, p. 481). Yet despite building up rapport with interviewees, qualitative researchers adopted the detached approach common in natural sciences in terms of the neutrality of the interviewer (Briggs, 1986, p. 21; Edwards & Holland, 2013, p. 15; Kvale, 2007, p. 21). Interviewers were not to “contaminate” or “distort” data by inserting their own view before or during the interview (Rogers, 1945, pp. 281, 282; Seidman, 1998, pp. 69, 73, 81).

At the same time, qualitative scholars emphasized that the interviewer must create an atmosphere that facilitates the acquisition of interviewees’ attitudes, understandings, and reasoning. Terms and phrases to describe this rapport include “friendship as a method” (Tillmann-Healy, 2003) and an “ethic of care” (Gilligan, 1982, 2014)

From around the 1980s, the aforementioned closeness between interviewee and interviewer with a vision to gain personal data from people in the form of stories and personal experiences came to be criticized as an exploitative tool of “doing rapport by faking friendship” (Duncombe & Jessop, 2012, p. 108ff). Feminist scholars were especially engaged in this debate (Kezar, 2003; Oakley, 1981; Stacey, 1988). Around the turn of the century, scholars also began weighing whether qualitative interview approaches were too focused on the experience of the interviewee at the expense of producing new knowledge (Atkinson & Silverman, 1997; Brinkmann, 2007; Curato, 2012; Kvale, 2006; R. C. Smith, 2003). New qualitative methods, thus, emerged that focused on co-constructing knowledge through a conversation or debate wherein the neutral stance of the interviewer was foregone (Gubrium & Holstein, 2003, pp. 70, 71; Talmy, 2010, p. 131).

In summary, the history of qualitative interviewing began as a response to the dominant positivist approach in the social and behavioral sciences. At its origins, qualitative interviewing focused on building rapport, maintaining researcher neutrality, and capturing the experience and perspective of the interviewee. A majority of academic disciplines maintain this understanding of qualitative interviewing. However, in recent years, a second wave of qualitative approaches emerged, which has shifted the focus from gleaning personal information in a neutral encounter toward engaging in dialogue and deliberation.

## Interviewing Styles

Scholars have used broad categorizations to delineate types of interviews in terms of the nature of the interview guide^4^ (Denzin, 2009; DiCicco-Bloom & Crabtree, 2006; Gorden, 1969; Mishler, 1986) or in terms of the overarching aim of the interview (Brinkmann, 2007; Curato, 2012; Kvale, 2007).

Labels used to define and categorize qualitative interview styles vary across scholars (Creswell, 2013; Kvale, 2007; Roulston, 2012), yet following Brinkmann’s classification, interviews can largely be grouped into two main categories^5^: doxastic and epistemic (Brinkmann, 2007). Doxastic comes from the Greek term for opinion or judgment, δόξα (doxa), whereas epistemic draws on the Greek notion of “true and scientific” knowledge or ἐπιστήμη (episteme; Peters, 1967, pp. 40-42, 59), which represents knowledge that has been tested or validated in an epistemic process. Doxastic interviews focus on the interviewee’s experience, attitudes, and understanding of the context (Brinkmann, 2007, p. 1117; Curato, 2012). Epistemic interviews emphasize the co-construction of knowledge between interviewer and interviewee, often through challenging one another (Brinkmann, 2007, p. 1124; Curato, 2012, p. 571). The distinctions made by Brinkmann (2007, 2016) and Curato (2011, 2012) on “doxastic” and “epistemic” interviews as well as Talmy’s table contrasting “interview as a research instrument” and “interview as social practice” inform the categorization presented in Table 1.^6^

**Table 1.** Overview of Qualitative Interviews.

Dimension	Doxastic interviewing	Epistemic interviewing
Purpose and interview concept (aim of interview)	“Interview as a research instrument for investigating . . . experience, beliefs, attitudes and/or feelings of respondents”^a^	“Interviews as social practice”—“data is collaboratively produced” “knowledge is co-constructed and cannot be contaminated”^a^
Interview relationship and information flow	Interviewer mostly in charge of questions, interviewee can decide how much to share largely unidirectional— information primarily from interviewee	Strives to be egalitarian, yet interviewer still often in charge of questions largely bi-directional— marked by debate and active exchange
Sharing of interviewer knowledge and experience	Interviewer is not supposed to share opinion, knowledge, or expertise in order not to bias outcome, yet sharing done in some styles (e.g., feminist)	Interviewer can provide expertise and knowledge as part of the dialogue, but it is not always clear, if this is done
Interview rapport	Very important	Less important
Role of interviewee	Respondent, informant, participant	Participant or interview partner; in some cases respondent
Role of interviewer	Listener, can probe but is not supposed to challenge	Engages in debate and tries to challenge interviewee
Data analysis	Analysis is more about the “what”^b^	Analysis is about the “what” and the “how”^b^

a Talmy (2010, p. 132); ^b^Gubrium and Holstein (2003).

## Doxastic Interviewing

Interviews that seek to gain an understanding of the interviewee’s experience and attitudes have several commonalities, namely, that the interviewer is expected to bracket or remove his or her attitudes, perceptions, and knowledge from the interview (Rogers, 1945; Seidman, 1998; Ulin et al., 2005). The interviewer acts as a facilitator tasked with eliciting information from the interviewee that may deepen the understanding of a subject matter with which the interviewee is intimately familiar by virtue of his or her lived experience (Moreira & Monteiro, 2009; Oakley, 1981; Svedhem, Eckert, & Wijma, 2013).

In doxastic interviewing, knowledge sharing is asymmetrical and dialogue is largely one way (Kvale, 2006, p. 484). The interviewer’s role is to ask questions and to probe for follow-up information. Compared to routine conversations, in doxastic interviews, “turn-taking is less balanced . . ., the ethnographer asks almost all the questions, the informant talks about her experience” (Spradley, 1979, p. 67). The interviewer’s role is that of an attentive listener who captures depth, nuance, and clarity regarding the interviewee’s experience by expressing interest, feigning ignorance, and asking similar questions repeatedly (Spradley, 1979, pp. 67-69). While probing on discrepancies is permitted, openly disagreeing with the interviewee or causing the interviewee discomfort is discouraged (Ulin et al., 2005, pp. 86, 88).

The interviewer also tries to record the interviewee’s position as accurately as possible “recognizing that a respondent’s distinct social location offers unique insight into a particular social phenomenon” (Mies quoted in Curato, 2012, p. 572). A range of interview styles that fall under the umbrella of doxastic interviews are described and critiqued in detail in the following including narrative, phenomenological, ethnographic, and feminist interviews.^7^ Reflexive and to some degree also feminist interviews show elements of both doxastic and epistemic interviews. Based on the overarching aim of doxastic interviews in understanding the interviewee and his or her experience, we have situated reflexive and feminist interviews in the doxastic field.

### Narrative Interviews, Oral and Life Histories

Narrative, biographical, or life history interviews originated in the field of sociology in the 1970s and 1980s. Narrative interviews are based on a person’s life experience and conducted to investigate the personal experience of important life episodes or key events (Atkinson, Bauer, & Gaskell, 2000; Rosenthal, 1993; Schütze, 1983). Such interviews may cover upbringing, schooling, work-related issues, or friendships and sexuality (Ritchie & Lewis, 2003, pp. 118-120) and are often used to investigate the effects of historical or political events such as wars and exile on life courses and life histories (Atkinson et al., 2000; Schütze, 1992).

In a narrative interview, the interviewer will listen attentively and let the interviewee recount his or her biography freely and without interruption. Only in the latter part of an interview or an interview series will the interviewer seek clarifications (Flick, von Kardoff, & Steinke, 2004; Rosenthal, 1993). Through narrative interviews, “a person has reconstructed the past to negotiate an ever-fluid process of identity construction” (R. C. Smith, 2003, p. 216).

### Phenomenological Interviews

Phenomenological interviews originated in the 1960s and go back to the philosophers Husserl and Schutz (Fontana, 2003, p. 55). In psychology, sociology, public health, and nursing, a phenomenological approach is common, and interviews are often conducted with people who share a health outcome, for example, living with HIV (Creswell, 2013, pp. 76, 104, 114) or a similar lived experience of a phenomenon, for example, involvement in sex-work (Moreira & Monteiro, 2009). The approach lends itself to exploring and understanding the issues patients or affected groups grapple with (Svedhem et al., 2013), or to identify factors for improving situations among affected groups (Östergård, Englander, & Axtelius, 2016).

An important concept within phenomenological interviews is “phenomenological reduction” or “bracketing,” that is, trying to set aside the interviewer’s own experience and assumptions with regard to a phenomenon in order not to impose this understanding on the data (Chan, Fung, & Chien, 2013, p. 1; Tufford & Newman, 2012).

### Ethnographic Interviews

Ethnographic interviews originated in social anthropological field methods and were employed mainly from the early 20th century in combination with participant-observation in a quest “to grasp the native’s point of view, his relation to life, to realize his vision of his world” (Malinowski, 1922, p. 25).

Ethnographic research consists of spending significant amounts of time with people to understand their culture, history, language, social customs, and behavior. Ethnographic interviews are, therefore, often unstructured, can happen at any time, and are accompanied by observation (Bernard, 2006, pp. 211-212; Spradley, 1979, pp. 4-5). Ethnographers also prefer the term *informant* to respondent, as an informant does not just answer questions but helps the ethnographer formulate questions that are culturally relevant—in that sense the informant is also the teacher (Spradley, 1979, pp. 25, 31f). Informants can also question the ethnographer (Evans-Pritchard, 1969, pp. 12, 13). Research questions are often adapted to be of greater relevance to the lives and concerns of informants (Spradley, 1979, p. 37).

### Feminist Interviews

Feminist interviews emerged from feminism and social justice movements in the second half of the 20th century and became more prominent from the 1970s. Feminist research set out to “correct both the *invisibility* and *distortion* of female experience in ways relevant to ending women’s unequal social position” (Lather, 1988, p. 571), and to give the marginalized and oppressed a voice (Collins, 2002; Mies, 1979). Lather argued that the feminist research approach should lead to empowerment and “contribute to change enhancing social theory” (Lather, 1988, p. 570).

In the early 1980s, feminist scholars like Oakley (1981) questioned the interview requirement of only eliciting information without giving information as an interviewer. Carrying out multiple interviews with women before and after they gave birth in London, Oakley recounted answering hundreds of questions by the women during her research (p. 42) and concluded that feminist research methods that place human interaction above often unattainable interview requirements should inform social science research (p. 58). Lather shares the sentiment that “neutrality is not only impossible but serves no real purpose” (Kezar, 2003, p. 400).

The feminist interviewing approach has emphasized agency among interviewees during interviews, stating that interviewees should be allowed to express themselves and their feelings freely, and interviewers should be active participants in the process (Fontana, 2003, p. 58). While feminist interviewers primarily focus on the experience of interviewees, they oppose the neutral stance of the interviewer and, thus, integrate epistemic elements.

### Reflexive Interviews

Studies of reflexivity, subjectivity, and auto-ethnography^8^ emerged in greater numbers in the 1970s and 1980s (Finlay, 2002). The approaches emphasize that researchers’ experiences, socialization, and world view influence data collection and interpretation and that it is necessary for researchers to talk about their experiences, interests, and presuppositions in order for others to understand the research perspectives and the conclusions drawn (Mruck & Breuer, 2003, p. 191f). There are no generally agreed guidelines for conducting reflexive interviews (Mruck & Breuer, 2003). Some scholars have conducted and analyzed a self-interview in addition to interviews with participants (Bolam, Gleeson, & Murphy, 2003), others have shared relevant personal details with interviewees during interviews (McNair et al., 2008) or focused on nonverbal communication including tension between interviewer and interviewees (Heizmann, 2003). The psychologists Russel and Kelly observed that the nature of the interview changed with increased interaction between interviewer and interviewee. “The roles of the interviewer and interviewee have become so blurred as to all but disappear” (Russell & Kelly, 2002, p. 8)—thus reflexive interviews contain elements more closely associated with “epistemic” interviews.

## Epistemic Interviewing

Brinkmann coined the term *epistemic* for interviews that are not primarily about the experience of the interviewee but are instead about constructing knowledge between interviewer and interviewee through an exchange of ideas (Brinkmann, 2007). Such interviews encourage the interviewee and interviewer to challenge one another in an attempt to understand underlying assumptions of what has been said (Tanggaard, 2007, p. 171), to bring in other perspectives, or to take the conversation to a higher level of knowledge (Curato, 2012, p. 571; Tanggaard, 2007, p. 171). Epistemic-type interviews usually refer to Socrates’ technique of challenging his dialogue partner (Brinkmann, 2007, p. 1125). Socrates was not primarily interested in the personal experience of his dialogue partner but in the understanding and reasoning of substantive issues. He would highlight inconsistencies in arguments and would stop only when a conclusion was reached (even if the conclusion entailed ἀπορία [aporia], or philosophical puzzlement). Through dialogue, definitions were often discarded and sometimes new definitions established (Brinkmann, 2007, p. 1127).

### “Active” Interviews

The term *active* or *Socratic* interviews was coined by Bellah in the 1980s (Bellah et al., 2008). In active interviews, the role of the interviewer is to “provoke responses by indicating—even suggesting—narrative positions, resources, orientations and precedents” (Gubrium & Holstein, 2003, p. 75). Bellah and his co-authors employed this interview approach in more than 200 interviews among middle-class Americans in the 1980s, persistently questioning interviewees to understand their position, reveal assumptions, and “to make explicit what the person we were talking to might have rather left implicit” (Bellah et al., 2008, p. 304).

Gubrium and Holstein compare an active interview to an improvisational performance, which transforms the way the interviewee is seen and treated from a “repository of opinions” and “wellspring of emotions” to a “productive source of knowledge” (Gubrium & Holstein, 2003, pp. 74, 75)

### “Elite” or Expert Interviews

Interviewing *elites* has become an essential part of journalism, particularly with live broadcasts (Clayman & Heritage, 2002). In the social sciences, Dexter can be seen as one of the pioneers of “elite” interviewing with his work with U.S. congressmen in the 1950s (Dexter, 2006; Kezar, 2003; Seidman, 1998), yet elite or expert interviews are often an integral part of political, pedagogical, and psychological research (Bogner, Littig, & Menz, 2014, p. 1).^9^

The method of constructing knowledge by challenging and being challenged seems to lend itself to conducting interviews with academics or specialists as they are used to inquiries and are less likely to take offense at being challenged (Kvale, 2007, p. 70). The main purpose of talking to experts is to gain a deeper or wider understanding of the issues to be researched. Bogner et al. distinguish between technical, process, and interpretive knowledge of experts (Bogner et al., 2014, p. 17.18) and some would argue that a structured interview guide might be less appropriate than letting the conversation flow (Kezar, 2003, p. 407), as experts are used to being in control and will often take the lead (Conkright in Seidman, 1998, p. 89), or take the liberty to co-design the interview (Curato, 2011, p. 268).

In expert interviews, interviewers may be regarded as inferior to their interviewee in terms of knowledge or position (Kezar, 2003, p. 409) and must identify strategies to earn respect often demonstrating knowledge on the subject matter (Curato, 2011; Kvale, 2007, p. 70; Richards, 1996). Scholars have recounted efforts made by both the interviewer and the interviewee to establish their rank and authority (Kezar, 2003; McDowell, 1998, p. 264f; K. E. Smith, 2006, p. 647). Yet there is no single method propagated for interviewing “elites” (Bogner et al., 2014, p. 3).

### Confrontational Interviews

Confrontational interviews—a term used by Kvale (2007)—seem to have come to the fore from the 1990s (Bourdieu, 1999; Tanggaard, 2007, 2008) Proponents of this approach to interviewing argue that, through a dialectic process where interviewees and interviewers may not agree on a particular meaning, a deeper understanding can be reached (Tanggaard, 2007). This interview technique breaks with two taboos of conventional qualitative interviews: The interviewer challenges the interviewee, and the interviewee may also challenge the interviewer. Kvale mainly cites Bourdieu and Bellah as representing confrontational interviewing (Kvale, 2007, pp. 75-76).^10^ We also include Tanggaard as a key representative of this interviewing technique. She argues for a widening of the term *qualitative interviews* to include “discourses crossing swords on meaning” (Tanggaard, 2007, p. 174) to allow disagreements and conflicting views regarding interview questions or the understanding of the issue, and she sees interviews as “negotiations of meaning” (p. 163).

### Deliberative Interview

Building on epistemic interviewing and developing this approach further by adapting the concept of deliberation to the interview situation, we propose a new interviewing style that we call the deliberative interview. The notion of employing deliberation in qualitative methods is not new. Deliberation has been defined by Fishkin as “discussing issues with others with different experiences, holding different views and representing varied and sometimes conflicting interests” (Fishkin, Luskin, & Jowell, 2000, p. 658). It has been used in group situations such as citizens’ juries, deliberative workshops, consensus conferences, deliberative polling, and deliberative mapping (Davies et al., 2003; Fishkin & Luskin, 2005; Scottish Government, 2009). Participants are given extensive information on a controversial issue to be discussed and are encouraged and guided to consult with experts and specialists to get in-depth information on the matter from different angles and perspectives before reaching a final group decision (Burchardt, 2014; Pidgeon, Demski, Butler, Parkhill, & Spence, 2014; Rychetnik et al., 2013). Informed deliberations may at times lead to a change of opinion of some participants (Abelson, Blacksher, Li, Boesveld, & Goold, 2013; Abelson et al, 2003; Davies et al., 2003; Fishkin & Luskin, 2005; Gregory, Hartz-Karp, & Watson, 2008), yet some scholars see the pre-occupation with “consensus” as counterproductive in conducting a true dialogue (G. Smith & Wales, 2000).

Curato suggested that deliberative elements could enhance Brinkmann’s epistemic interviews (Curato, 2012) but did not detail how one would prepare and conduct such an interview, which we call “deliberative interview.” Applying deliberation to the interview situation will require that both interviewer and interview partner^11^ reason together, have about the same speaking time,^12^ and that they can question, even challenge one another in the joint search for a better understanding or more suitable solutions to the issues at stake. The main objective in the interview situation is not to reach consensus but rather to produce workable solutions with regard to the issue or problem to be discussed. As with group deliberation methods, an in-depth and balanced background briefing before the interview or dialogue begins is an essential element of a deliberative interview, as the know-ledge level of interviewer and interviewee on the topic to be discussed should not differ considerably.^13^ Since many interview partners are not familiar with an interview situation in which their point of view is questioned and in which they are expected to challenge the interviewer, a thorough methodological briefing is necessary. This diverges considerably from other interview styles where neither thematic nor methodological briefing takes place.

We envisage this approach to qualitative interviewing to be of particular use for academic disciplines seeking to generate practical solutions to complex issues. Our own interest is primarily in the field of public health and ethics, exploring questions such as the type of informed consent and ethical oversight required for health improvement initiatives in which the risk of causing harm is often minimal (Faden, Beauchamp, & Kass, 2014; Finkelstein et al., 2015; Fiscella, Tobin, Carroll, He, & Ogedegbe, 2015; Hutton, Eccles, & Grimshaw, 2008; Kilama, 2010) or how to prioritize limited budgets for health with regard to access to diagnostics and treatment in resource-limited settings (Anderson et al., 2006; Eaton et al., 2014; Ubel, DeKay, Baron, & Asch, 1996). Yet we believe that that this approach to qualitative interviewing will be valuable across many disciplines, for example, in psychology or sociology, where it can be used to elucidate how humans develop knowledge in social situations, and to explore issues including (mental) health and social justice.

**Figure 1.**
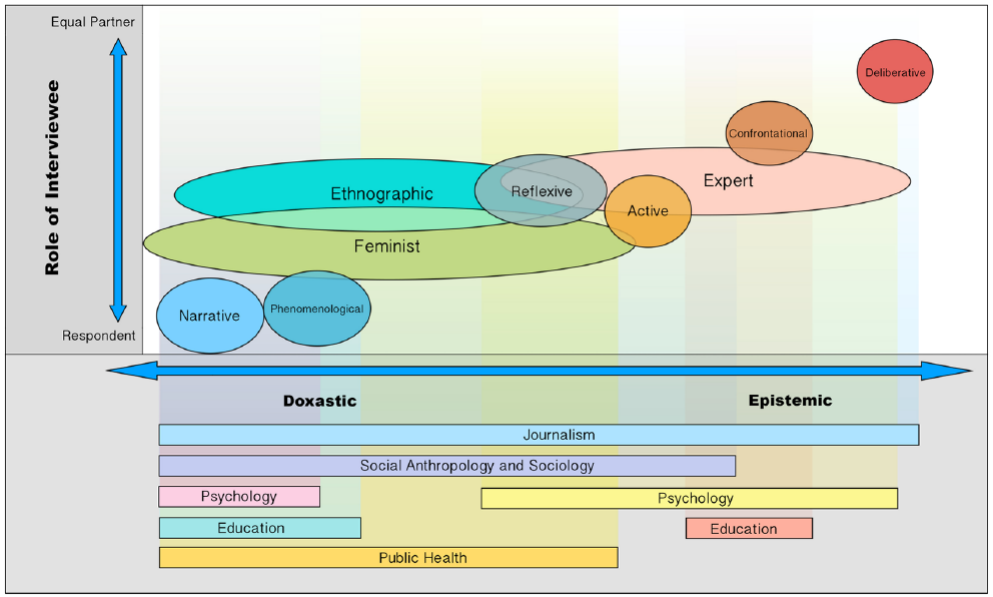
Typology of Qualitative Interview Styles. *Note.* A presentation of interview styles by role of interviewee, aim of interview and academic discipline that typically employs the style.

Figure 1 visualizes the doxastic and epistemic interview styles described earlier in terms of their usage in academic disciplines as well as the degree to which the interview style focuses on experience or constructing knowledge (*x*-axis) and reflects the role of the interviewee as respondent answering questions or equal interview partner who can also challenge the interviewer (*y*-axis).

Interview styles are positioned on the *x*-axis according to the purpose of the interview. Doxastic interviews are toward the left of the *y*-axis, epistemic interviews are positioned further to the right of the *x*-axis. The role of the interviewee as respondent (answering questions posed by the interviewer) or interview partner (who can question or challenge the interviewer) are used as the dimension of difference across interview styles shown on the *y*-axis. We chose these dimensions because they capture important differences in the practical conduct of the interviews.

While narrative and phenomenological interviews focus clearly on the experience of the interviewee with the interviewee responding to questions, confrontational interviews expect the interviewee to challenge the interviewer, thus treating the interviewee more as an equal partner and predominantly focus on examining an issue from various perspectives and co-constructing knowledge between interviewee and interviewer. This is even more pronounced in deliberative interviews where the interviewee is tasked to find a solution together with the interviewer and, thus, generates new knowledge and insights through dialogue as equal partners.

The two dimensions are related in that the more epistemic interviews are expected to gain in equality regarding the role of interviewee and interviewer, yet as some interview examples show, doxastic interviews can also lead to the interviewee challenging the interviewer (Evans-Pritchard, 1969), and some epistemic interviews such as examples of active interviews do not show that the interviewee questions or challenges the interviewer—the challenging seems to be done by the interviewer only (Bellah et al., 2008, pp. 304-305; Bourdieu, 1999). The graph, therefore, represents a general tendency rather depicting each interview within a given style. Deliberative interviews represent the most epistemic interview and the most equal in terms of interviewee’s and interviewer’s role.^14^

The interview style bubbles vary in size depending on whether a style can incorporate others as is the case with ethnographic or feminist interviews, which can take the form of narrative, reflexive, or expert interviews. Ethnographic and feminist styles are more deeply anchored in the doxastic field yet can contain epistemic elements. Expert interviews can be incorporated into a number of interview styles, yet have a tendency to co-construct knowledge between the expert interviewee and the interviewer, and to treat the expert as an interview partner who can challenge the interviewer or express doubts on particular research methods or questions. The graph also acknowledges Brinkmann’s (2013) statement that interviews may consist of both “doxastic” and “epistemic” elements so that these terms are to be understood on a scale or on axes (pp. 156, 162).

The predominant usage of a particular interview style in various academic disciplines are listed below the *x*-axis. While public health and social anthropology/sociology primarily aim at understanding experience and behavior with the latter also using interview styles that co-construct knowledge, psychology and education use doxastic and epistemic approaches, and journalism covers all interview styles and approaches.

## Interview Characteristics

As the interview approaches and styles suggest, debates among qualitative researchers continue regarding the importance of rapport and power relations, the amount of talking time and interaction, and the process in qualitative interviewing and how to interpret the data. Discussed in the following (and in Table 2) is an overview of characteristics across interview styles.

**Table 2.** Characteristics of Qualitative Interview Styles.

Doxastic: Focus on interviewee’s experience					Epistemic: Focus on co-constructing knowledge				
Characteristics	Narrative	Phenomenological	Ethnographic	Feminist	Reflexive	Active	Elite/expert	Confrontational	Deliberative
Purpose	“Tell stories of individual experiences” (Creswell, 2013, p. 104)	“Describe the essence of a lived phenomenon” (Creswell, 2013, p. 104)	Describe and explain cultural differences (Spradley, 1979)	Describe diverse realities of women and marginalized groups (Mies, 1979); “empower the researched” (Lather, 1988, p. 570)	“Reflect on subjective nature of knowledge production” (Mruck & Breuer, 2003, p. 194)	“Uncover assumptions” (Bellah, Madsen, Sullivan, Swidler, & Tipton, 2008, p. 304)	Discover technical, process-related or interpretive-evaluative knowledge (Bogner, Littig, & Menz, 2014)	“Negotiation of meaning” through “clash of discourses” (Tanggaard, 2007, p. 161)	Co-construct knowledge
Interview rapport	Very important	Very important	Important, yet choice of good informant seems more important (Spradley, 1979)	Extremely important for overcoming differences (yet also critiqued as overrated; cf. Lyons & Chipperfield, 2000)	Important	Less important	Less important	Less important	Less important
Interview relationship	Interviewer in charge of questions, yet interviewee decides how much and what experience to reveal	Interviewer in charge of questions, yet interviewee decides how much and what information or experience to reveal	Interviewer mostly in charge of questions, yet interviewer often “pupil” of informant	Strives to be egalitarian, yet interviewer still in charge	Strives to be egalitarian, yet interviewer still in charge	Strives to be egalitarian, yet interviewer still in charge	Interviewer mostly in charge of questions, yet expert interviewee often holding higher position	Strives to be egalitarian, yet interviewer still in charge, cf. examples (Tanggaard, 2007)	Egalitarian - interviewer and interviewee can exchange roles during interview
Sharing of interviewer knowledge/experience	No sharing, as this may influence interview	No sharing, as this may influence interview	Sharing depends on curiosity of informants (Evans-Pritchard, 1969)	Sharing common (referred to as self-disclosure)	Sharing common (McNair, Taft, & Hegarty, 2008) and documented, analyzed, and presented in write- up	Unclear	Variable-interviewer often demonstrates knowledge to balance higher social position of interviewee	Unclear	Interviewer knowledge shared throughout interview
Role of interviewer	Listener, little interference, probes, seeks full under-standing, does not challenge interviewee	Listener, little interference, probes, seeks full understanding, does not challenge interviewee	Probes in order to understand meaning; might challenge, if it helps in understanding issues but mostly lets informant talk	Active listener	Interviewer is part of the process	Tries to clarify, even challenge interviewee in order to better understand reasons for behavior	Tries to clarify and challenge	Tries to clarify, even challenge interviewee in order to better understand concepts	Challenges interviewee as much as possible in order to find a well-reasoned solution
Role of interviewee	Respondentshares his or her experience, does not challenge interviewer	Respondent/participantSeidmann/Creswell use “participant” but agency limited; shares his or her experience; does not challenge interviewer	Informanthelps interviewer to ask pertinent questions and understand culture	Respondent/participantfeminist scholars talk about participants, yet interviewee still largely shares his or her story	Participantpossibility to ask interviewer questions (Finlay, 2002; McNair et al., 2008)	Respondent/participantengages in a “Socratic” type of dialogue, yet questions mainly asked by interviewer	Participantcan challenge interviewer and even co-design data-gathering strategy (Curato, 2011)	Participantcan challenge interviewer, interviewing questions, and present his or her view	Interview partner can challenge and ask questions as interviewer can
Data analysis	Analyzing data for stories, developing themes, often using a chronology (Creswell, 2013, p. 191)	Analyzing data for significant statements, meaning units, textual and structural description, and description of the “essence” (Creswell, 2013, p. 191)	Analyzing data through description of the “culture-sharing group” and emerging themes (Creswell, 2013, pp. 191, 197)	Analyzing interactions and taking context into account (DeVault & Gross, 2011)	Analyzing data in terms of personal bias (Ahern, 1999)	Analyzing interview data to show that interview responses are produced in the interaction between interviewer and interviewee (Gubrium & Holstein, 2003)	Analyzing data for significant statements, meaning units as well as additional knowledge on research theme	Analyzing negotiations of meaning in the interview between interviewer and interviewee (Tanggaard, 2007)	Analyzing how final position is reached and if change of opinion could be noticed Process and outcome important

*Note.* More recently, some feminist scholars have argued that “any method can be made feminist” (quoted in Creswell, 2013, p. 30), yet it seems important to understand the approach that feminist scholars have developed.

### Interview Rapport

In doxastic interviews, as noted earlier, building a relationship with the interviewee is essential for the interviewee to open up and reveal personal information. Some scholars even suggest that “good interviewing will draw out from interviewees what they would be reluctant to tell most people” (Rosenblatt, 2003, p. 229).

In epistemic interviews, rapport seems to be less important, as the issues to be discussed are usually less personal. Yet it will hardly be possible to conduct interviews without establishing some working relationship between interviewer and interviewee, which may include agreeing that it is fine to disagree in the dialogue.

### Mutuality and Symmetry Including Interview Relationship

Analyzing interviews in terms of speaking time, interviewee statements, and knowledge gleaned on a subject, highlights marked differences across styles. Kvale sees interviews as asymmetrical and views “the term *interview dialogue* . . . a misnomer” (Kvale, 2006, p. 483).

Corbin and Morse categorized the interviewing approaches in terms of roles, power relations, and interaction between “researcher” and “participant” (Corbin & Morse, 2003, p. 340). They noted that in unstructured interviews, the agenda is set by the interviewee who also largely controls the degree of sharing personal information. The interaction is depicted as one-way: the interviewee speaking, the interviewer listening. With regard to semistructured interviews, the agenda setting is done by the interviewer through the questions asked, yet the interviewee can withhold information and is, therefore, seen by Corbin and Morse as having control over the interaction. The direction of interaction is depicted as two-directional (Corbin & Morse, 2003, p. 340).

While one might dispute this with regard to doxastic interviews where the direction of interaction still seems to be largely a “one-way dialogue” (Kvale, 2006, p. 484), with regard to epistemic interviews, the interaction would ideally be going in both directions. However, the concrete examples given for active, elite, and confrontational interviews still suggest that although both interviewer and interviewee are active participants in the interview, it is still mostly the interviewee who shares his or her understanding. An egalitarian relationship does not seem to have been reached yet.^15^

### Data Analysis

While methods vary, in most instances across styles, it is the researcher who has the final say about how to interpret data in analyzing and publishing the results from interviews (Stacey, 1988, p. 23). In terms of epistemic interviews, the analysis is “in” the conversation Brinkmann (2007) and, thus, a result of the engagement in the interview of both interviewer and interviewee. In this sense, Platonic dialogues^16^ represent an archetypal form of conversation wherein the discussion embodies an analysis of the themes from within the conversation. In other approaches to qualitative interviewing, analysis in terms of “de-constructing” the exchanges to show outcome and process of “meaning-making” (Gubrium & Holstein, 2003, p. 79) is mostly undertaken after the interview interaction is concluded. In deliberative interviews, an analysis of a change of opinion of either interviewee or interviewer will also be made analogous to deliberative group methods (cf. Pidgeon et al., 2014, p. 13610).

## Concluding Remarks

In providing an overview of disciplinary histories, comparisons, and critiques of common interview styles, we have built on Brinkmann’s distinction of doxastic *versus* epistemic interview types to develop a typology of interviews that position existing styles along two continua—from a focus on understanding the interviewees’ experiences, behaviors, and context to co-constructing knowledge, and from the role of the interviewee as a respondent to an equal partner.

Building on Curato’s work (2012), we have argued that there is need for an expansion of epistemic-type approaches, particularly in academic fields that are concerned with complex ethical and moral issues and, thus, require recommendations and solutions based on the active and informed engagement of the interviewee with the interviewer for joint deliberation and co-construction of new knowledge and insights. We have also provided further details on how to conduct a deliberative interview and have shown that a methodological and issue-focused briefing of the interviewee is an essential part. This briefing phase before the interview starts is unique to deliberation and diverges considerably from conventional doxastic and epistemic interviews.

Our critique has also revealed discrepancies between epistemic rationales for interviews and the concrete methods and approaches used, which are still largely conducted in the doxastic mode with the interviewer asking questions, the interviewee answering rather than engaging in dialogue and deliberation. Deliberative interviews, as we develop them in this text, would allow a maximum feasible level of deliberative interaction for the joint creation of a mutual understanding and evaluation of complex issues and questions seeking normative or practical answers.

## Notes

1. Different terms are used by different scholars to describe the person with whom one conducts an interview, yet the terms do not always reflect the role they are assigned but seem to be largely determined by the scholar’s academic discipline. While anthropologists routinely speak of “informants” (Spradley, 1979), psychologists often use the term “subject” (Kvale, 2006), public health specialists tend to state “respondents” (Baicker et al., 2013), others prefer to speak of “interviewees” (Randall, Prior, & Skarborn, 2006) or “participants” (Seidman, 1998). However, examples given often do not show much agency by the interviewee who mainly answers the questions posed by the interviewer, thus fulfilling more the role of “respondent.” Action theorists often speak of “co-researchers” (Seidman, 1998, p. 8), although this seems to suggest that both agree on the research topic together, which is rarely the case. We use the relatively neutral term *interviewee* in this paper, unless the interviewee has the same freedom to question and challenge, then we prefer to speak of interview partners.

2. We would like to acknowledge Curato (2012), who without using the term *deliberative interview* suggested to enhance epistemic interviews by using a deliberative model. While Curato gave the example of an “off the record” dialogic interview based on deliberation, we are introducing a new interview style called “deliberative interview.”

3. Kuhn shaped the debate about paradigms in the 1960s arguing that “anomalies” eventually lead to a paradigm shift (Kuhn, 1970, p. 101; Wise, 2016, p. 33). Kuhn (1970) also spoke of paradigms as a “disciplinary matrix” (p. 182-184) and in this sense, one can see how different academic disciplines subscribed more to one “matrix” than another. For the social sciences, Kuhn’s ideas led to the aspiration to apply similar paradigms as were used in the natural sciences, although in the 1970s, there was still an ongoing debate as to what extent paradigms were applicable to the social sciences at all (Eckberg & Hill, 1979).

4. That is, unstructured, semistructured, and structured interviews. Structured interviews with their standardized format for questions and answers can be used for better comparison and, thus, align more closely with a quantitative perspective. Some scholars, therefore, restrict the term *qualitative interview* to semi- or unstructured interviews (Ritchie & Lewis, 2003; Roulston, 2012). Unstructured interviews originated in social anthropological research where the method was combined with participant observation, yet the application of this interview method in studies without extended periods of fieldwork is limited. Interviews are usually adapted from unstructured to semistructured when they become the main method for collecting data (DiCicco-Bloom & Crabtree, 2006, p. 315).

5. Some scholars such as Alvesson (Alvesson, 2003, p. 16) or Qu and Dumay (Qu & Dumay, 2011, p. 242) have categorized interviews into three categories, yet differed in their categorization of what we call “epistemic.” We will use Brinkmann’s classification, keeping in mind that any categorization is a simplification.

6. Table 2 will provide more information about concrete interview styles.

7. It should also be noted that some interviewers use a combination of interview styles, for example, some feminist scholars have made use of ethnographic interviews (Stacey, 1988), and Seidmann combined life-history interviewing with phenomenology (Seidman, 1998, p. 9).

8. Mainly employed in social anthropology.

9. We acknowledge the problematic term *elite*, which suggests “powerful elites” juxtaposed with “powerless others,” cf. Reismann and Woods (quoted in K. E. Smith, 2006, p. 645). We, therefore, favor the term *expert*.

10. Bellah, Madsen, Sullivan, Swidler, and Tipton (2008) asked persistent questions on the responsibility of the interviewee toward other family members and Bourdieu in an interview with two urban youth in a housing project pointed out that the strong reactions they got from residents were also due to them making noise late at night (Bourdieu, 1999, pp. 64-65).

11. We will use the term *interview partner* for “deliberative interviews” to emphasize that the interviewee is a partner in dialogue who holds equal standing in relation to the interviewer.

12. While the exact speaking time depends on many factors, personalities included, this interview approach will also enable the interviewer to take a more active part in reasoning.

13. The background briefing will be more extensive, where initial knowledge levels differ greatly.

14. It should also be noted that in epistemic interviews, it may sometimes be difficult to achieve equality due to pre-conceived roles of interviewee and interviewer, which are often difficult to overcome in an interview situation. To achieve greater equality in deliberative interviews, a thorough methodological briefing before the interview seems to be essential.

15. Examples by Bellah et al. (2008) and Bourdieu (1999) show that it is the interviewer who questions persistently or provides a different view to that of the interviewees, yet the interviewees still largely answer questions rather than challenge the interviewer. Also, the examples by Brinkmann (2007, 2013) seem to mainly provide different perspectives, which the interviewer explores with the interviewees rather than addressing the co-construction of knowledge. Examples by Tanggaard (2008) are mostly in terms of a different interpretation of concepts between interviewer and interviewee, for example, “learning” (p. 22)

16. Whether the dialogues actually took place or were used to demonstrate reasoning is of no consequence here.
